# FKRR-MVSF: A Fuzzy Kernel Ridge Regression Model for Identifying DNA-Binding Proteins by Multi-View Sequence Features via Chou’s Five-Step Rule

**DOI:** 10.3390/ijms20174175

**Published:** 2019-08-26

**Authors:** Yi Zou, Yijie Ding, Jijun Tang, Fei Guo, Li Peng

**Affiliations:** 1 School of Internet of Things Engineering, Jiangnan University, Wuxi 214122, China; 2 Engineering Research Center of Internet of Things Applied Technology, Ministry of Education, Wuxi 214122, China; 3 School of Electronic and Information Engineering, Suzhou University of Science and Technology, Suzhou 215009, China; 4 Department of Computer Science and Engineering, University of South Carolina, Columbia, SC 29208, USA; 5 School of Computer Science and Technology, College of Intelligence and Computing, Tianjin University, Tianjin 300350, China

**Keywords:** DNA-binding proteins prediction, fuzzy kernel ridge regression, multiple kernel learning, feature extraction, protein sequence

## Abstract

DNA-binding proteins play an important role in cell metabolism. In biological laboratories, the detection methods of DNA-binding proteins includes yeast one-hybrid methods, bacterial singles and X-ray crystallography methods and others, but these methods involve a lot of labor, material and time. In recent years, many computation-based approachs have been proposed to detect DNA-binding proteins. In this paper, a machine learning-based method, which is called the Fuzzy Kernel Ridge Regression model based on Multi-View Sequence Features (FKRR-MVSF), is proposed to identifying DNA-binding proteins. First of all, multi-view sequence features are extracted from protein sequences. Next, a Multiple Kernel Learning (MKL) algorithm is employed to combine multiple features. Finally, a Fuzzy Kernel Ridge Regression (FKRR) model is built to detect DNA-binding proteins. Compared with other methods, our model achieves good results. Our method obtains an accuracy of 83.26% and 81.72% on two benchmark datasets (PDB1075 and compared with PDB186), respectively.

## 1. Introduction

The interaction between DNA and protein exists in various tissues of the living body. For example, DNA–protein interactions during many activities such as DNA replication, DNA repair, DNA packaging, DNA modification, and viral infection. The study of DNA binding residues in DNA–protein interactions facilitates a comprehensive understanding of the mechanisms of chromatin recombination and gene-regulated expression. The methods of detecting DNA-binding proteins are mainly deployed by biochemistry and physical chemistry methods. However, wet experiment-based methods are both time and money consuming.

The protein information of 3D structures or their complexes is important for drug design. X-ray crystallography is expensive and time-consuming [1,2,3]. Lots of sequence-based information, such as PTM (posttranslational modification) sites in proteins [4,5,6,7,8,9], DNA-methylation sites [10], protein–drug interaction in cellular networking [11], protein–protein interactions [12] and recombination spots [13], have been predicted by sequential tools such as Pseudo Amino Acid Composition (PseAAC) [14] and Pseudo K-tuple Nucleotide Composition (PseKNC) approach [15]. Bioinformatics has played important roles in the development of novel drugs.

Computational methods based on Machine Learning (ML) have been developed to predict DNA-binding proteins. Currently, ML technology is playing key roles in lots of biological field, including prediction of DNA methylcytosine sites [16,17], O-GlcNAcylation sites [18], potential disease-associated microRNAs [19,20], protein remote homology [21], protein subcellular localization [22], electron transport proteins [23] and analyzing microbiology [24] et al. The computational methods can be classified into two types of methods: sequence-based models and a structure-based models.

The sequence-based methods extract features from protein sequences and employ ML to build predictive models. PseAAC and Support Vector Machine (SVM) [25] were used to construct a model for identifying DNA-Binding Proteins [26]. Kumar et al. [27] used Position Specific Scoring Matrix (PSSM) of protein sequences to develop an SVM classifier called DNAbinder. The PSSM describes proetin sequences. PSI-BLAST [28] can calculate PSSM for target protein. Liu et al. [29] proposed iDNAPro-PseAAC model, which employed PseAAC and PSSM features. Wei et al. [30] used local PSSM features to represent local information of proteins. Sequence-based approachs can implement large-scale predictions.

Structure-based models employ structure features to predict DNA-binding proteins. Compared with sequence-based methods, structure-based models achieve better performance. The main reason is that 3D structure of proteins determine the shape and surface area of the protein. Nimrod et al. [31] used the average surface electrostatic potentials of the protein to build a Random Forest (RF) model to predict DNA-binding proteins. Due to the known structures being less than sequences, the structure-based models can not predict all proteins.

In recent publications [32,33,34,35] and two review papers [36,37], researchers developed useful predictors for bioinformatics. Many methods obeyed a rule, called Chou’s five-step rule. This rule contains five steps: (1) a benchmark dataset is constructed to train and test the predictive models; (2) the selected samples should truly reflect their correlation of the target; (3) the prediction problem can be solved by a powerful algorithm; (4) the cross-validation tests are performed to evaluate the performance of the methods; (5) building a web-server for the predictive model. The above rule is clear in logic, and completely transparent in operation. This rule can easily repeat the reported results by other researchers and is very convenient for the experimental scientists. Our method is also based on Chou’s five-step rule.

To avoid losing the sequence–pattern information of proteins, the PseAAC [14,36,38] was proposed by Chou. Chou’s general PseAAC [36] has been widely used to extract features from sequence and PSSM of protein. In addition, a useful web-server called “Pse-in-One2.0” [39,40] has been established. The server can extract feature vectors for DNA/RNA and protein/peptide sequences. We also emply Pse-in-One2.0 to extract features from protein sequences.

In this study, we propose a novel model via a Fuzzy Kernel Ridge Regression model based on Multi-View Sequence Features (FKRR-MVSF) to predict DNA-binding proteins. The multiple sequence features are extracted and constructed to multiple kernels, respectively. Next, a Multiple Kernel Learning (MKL) algorithm linearly weights these kernels. Fuzzy membership scores of each training sample are calculated by an integrated kernel. Finally, Fuzzy Kernel Ridge Regression (FKRR) is trained to predict DNA-binding proteins.

## 2. Results

To evaluate our proposed method (FKRR-MVSF), two benchmark datasets of DNA-binding proteins are employed in our study. First of all, we analyze the performance of different features. Then, our model is compared with other methods via a Jackknife test. Finally, an independent test set is used to test the robustness of FKRR-MVSF.

### 2.1. Data Sets

In our study, two benchmark datasets (PDB1075 and PDB186 datasets) are used to test our predictive model of DNA-binding proteins. PDB1075 and PDB186 were collected from the Protein Data Bank (PDB) [41]. Liu et al. [26] randomly extracted non-DNA-binding and DNA-binding proteins from the PDB database. The similarity of any two sequences does not exceed 25%. A total of 525 DNA-bind proteins and 550 non-DNA-binding proteins form the PDB1075 dataset. PDB186 dataset [42] contains 93 DNA-bind and 93 non-DNA-bind proteins. Table 1 lists the information of the two benchmark data sets.

### 2.2. Measurements

Accuracy (ACC), Sensitivity (SN), Specificity (SP) and Matthew’s Correlation Coefficient (MCC) are used to evaluate the performance of predictive model. These coefficients are calculated as follows:

(1a) $$\begin{array}{cc} ACC & =1-\frac{N_{-}^{+}+N_{+}^{-}}{N^{+}+N^{-}} \end{array}$$

(1b) $$\begin{array}{cc} SN & =1-\frac{N_{-}^{+}}{N^{+}} \end{array}$$

(1c) $$\begin{array}{cc} Spec & =1-\frac{N_{+}^{-}}{N^{-}} \end{array}$$

(1d) $$\begin{array}{cc} MCC & =\frac{1-(\frac{N_{-}^{+}}{N^{+}}+\frac{N_{+}^{-}}{N^{-}})}{\sqrt{\left(1+\frac{N_{+}^{-}-N_{-}^{+}}{N^{+}}\right)\left(1+\frac{N_{-}^{+}-N_{+}^{-}}{N^{-}}\right)}} \end{array}$$

where $N^{+}$ and $N^{-}$ are the total number of positive and negative samples, respectively. $N_{+}^{-}$ and $N_{-}^{+}$ are the number of false positive and false negative, respectively. And Area Under ROC curve (AUC) is also an effective evaluation method for binary classification.

### 2.3. Performance Analysis of Different Features on the PDB1075 Data Set

The single type feature can not fully describe the properties of a protein, so we build the predictive model with multi-view sequence features to represent the protein. We test (Jackknife test evaluation) these features (kernels) on the PDB1075 dataset, as shown in Table 2. The PSSM-based features (PSSM-AB and PsePSSM feature) achieve better performance than non-PSSM (MCD and NMBAC feature) single features. The performance (MCC) of MCD, NMBAC, PSSM-AB and PsePSSM feature are 0.4139, 0.4564, 0.5113 and 0.5886, respectively. In addition, mean weighted kernels (KRR) combines the above 4 kernels (features) via average weight and obtains better performance (MCC: 0.6398) than single feature. Compared with mean weightes (KRR), MKL (KRR) achieves a higher value of MCC (0.6439). FKRR weighs training sets by fuzzy membership, which can filter outliers. So, mean weights (FKRR) (MCC: 0.6554) and MKL (FKRR) (MCC: 0.6664) are both better than KRR because of using multiple kernel information and fuzzy membership. Moreover, MKL (FKRR) achieves a better MCC of 0.6664.

**Figure 1:**
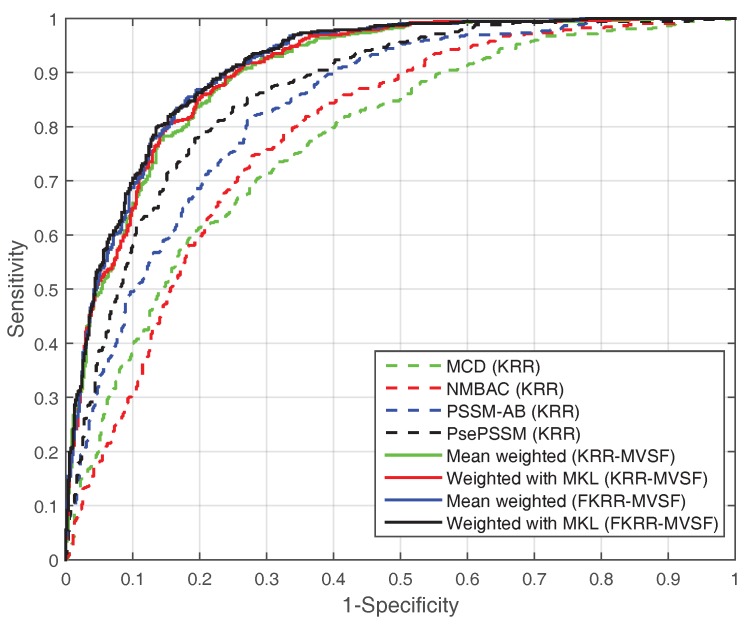
The ROC curve of different kernels (features) on the PDB1075 dataset (Jackknife test).

In addition, we test the SVM model with different features on the PDB1075 dataset. In Table 2, the performance (MCC) of SVM (with MKL, MCC: 0.6568) is better than KRR (with MKL, MCC: 0.6439). However, the MCC (0.6568) of SVM (with MKL) is slightly lower than FKRR (with MKL, MCC: 0.6664). The reason may be the fuzzy membership for building predictor. The ROC curve also reflects the excellent performance of MKL (FKRR) in Figure 1. Our method (FKRR-MVSF) employs MKL and FKRR to build a final predictor for DNA-binding proteins.

**Figure 2:**
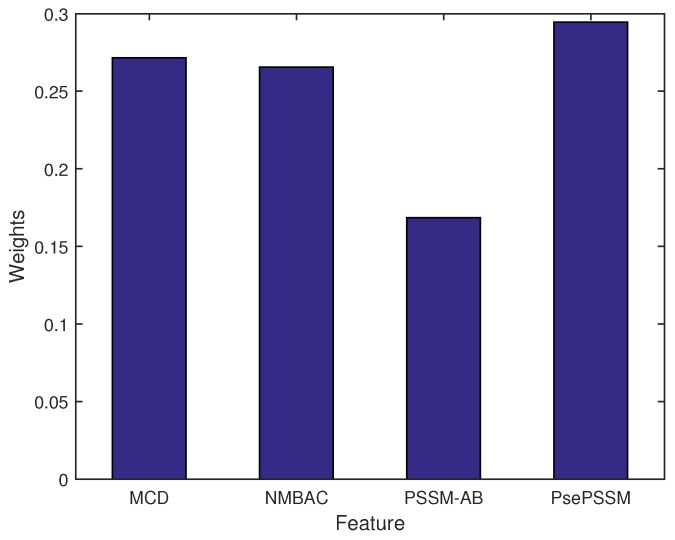
The weights of different kernels (features).

Figure 2 shows the weight of each feature. The highest weight of feature is PsePSSM, which has a similar trend of their single feature performance. To reduce bias of features, the MKL algorithm can estimate the optimal weights of features.

We test our method and other existing methods on the PDB1075 dataset. Table 3 lists the results of comparison between our method and other methods. PseDNA-Pro [26], IDNA-Prot|dis [29], IDNA-Prot [43], DNAbinder [27], DNA-Prot [44], iDNAPro-PseAAC [45], Local-DPP [30], Adilina’s work [46] and Kmer1+ACC [47] are benchmark methods. And IDNA-Prot|dis (MCC: 0.54), PseDNA-Pro (MCC: 0.53) iDNAPro-PseAAC (MCC: 0.53) and Local-DPP (MCC: 0.59) obtain better performance. Our proposed model (FKRR-MVSF) obtains best MCC (0.67) on the PDB1075 data set.

### 2.4. Performance on an Independent DataSet of PDB186

In order to evaluate the generalization performance of predictive models, FKRR-MVSF and other methods are also tested on the independent dateset (training set is PDB1075). The results are shown in Table 4.

Our method (FKRR-MVSF) achieves 81.7% of ACC, 0.676 of MCC and 98.9% of SN. In MCC, FKRR-MVSF is better than Local-DPP (MCC: 0.625), DBPPred (MCC: 0.538), MSFBinder [48] (MCC: 0.640), Adilina’s work (MCC: 0.670) and iDNAPro-PseAAC (MCC: 0.442).

## 3. Discussion

To improve the performance of predicting DNA-binding proteins, we employ an MKL algorithm and fuzzy-based model to integrated different features and further handle the outliers, respectively. There are many ways in machine learning to avoid overfitting and generating skewed models caused by outliers, e.g., adjustment of the cost value in SVM. For different training samples, the parameter of cost should be different. Different samples have different contributions to the model. In Table 2, the performance (MCC: 0.6664) of fuzzy-based models (FKRR with MKL) is better than non-fuzzy models (KRR with MKL, MCC: 0.6439).

Compared to other single kernels, the PsePSSM-based kernel achieves the highest weight and highest value of MCC (0.5886). MKL could integrate multiple information of sequence. Our method (KRR with MKL) also achieves better performance of MCC (0.6439) than a single kernel model on the PDB1075 dataset. In addition, the performance of KRR with MKL (MCC: 0.6439) is better than KRR with mean weights (MCC: 0.6398) under PDB1075 dataset.

On the independent test dataset, our method (FKRR with MKL) also achieves better MCC (0.676). MSFBinder (SVM) [48] is a two-layer model with SVM. MSFBinder (SVM) also employed several features to build a predictive model. The generalization performance of FKRR (withe MKL) is better than MSFBinder (MCC: 0.640) on an independent test set (PDB186). The above two models are similar. The main reason of different results is that the parameter *C* of FKRR is different for each train sample. Fuzzy membership may reduce the effect of some noise samples in the model.

## 4. Materials and Methods

**Figure 3:**
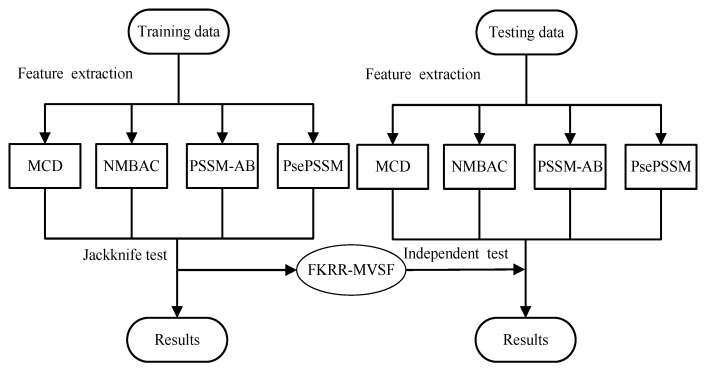
The process of DNA-binding protein prediction.

**Figure 4:**
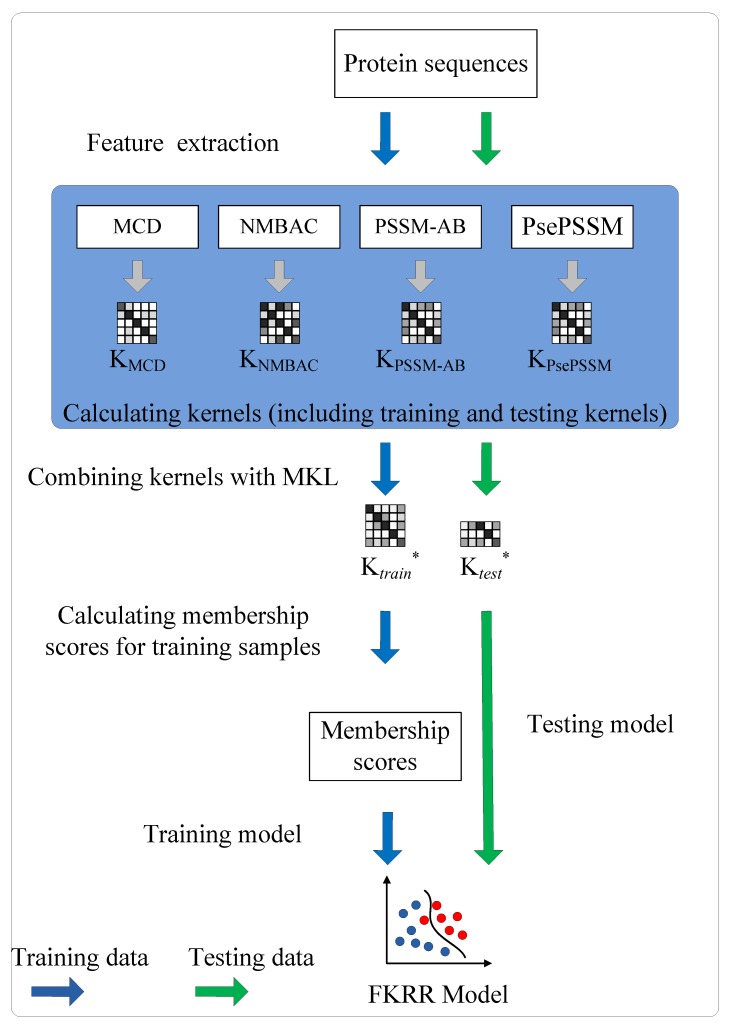
The process of FKRR-MVSF.

The prediction of DNA-binding proteins can be regarded as a task of binary classification. The protein can be represented by some feature vectors. The DNA-binding proteins and non-DNA-binding proteins are labeled as +1 (positive samples) and −1 (negative samples), respectively. We construct a Fuzzy Kernel Ridge Regression model based on Multi-View Sequence Features (FKRR-MVSF) to determine whether a protein binds to DNA. We employ Normalized Moreau–Broto Auto Correlation (NMBAC) [49,50], PSSM based Average Blocks (PSSM-AB) [51], Multiple-scale Continuous and Discontinuous descriptor (MCD) [52] and PsePSSM algorithms to extract four types of PSSM-based features. Radial Basis Function (RBF) is used to build four types of kernels from the above four kinds of features. In our study, the MKL algorithm is employed to calculate the weights of kernels and to combine four kernels. Then, a membership score is estimated for each training sample. Finally, a fuzzy kernel ridge regression model for identifying DNA-binding proteins is constructed via membership scores and a combined kernel. The framework of proposed method is showed in Figure 3. In the literature [13,33], the researchers have made good use of flowcharts to describe the main framework of their methods. In our work, we employ Figure 4 to describe the flow of our model. Firstly, we extract four types of feature from a sequence. Then, Radical Basis Function (RBF) is used to build four kernels. These kernels are conbined by MKL. Finally, combined kernel and training labels are employed to construct the FKRR model and predict new samples.

### 4.1. Feature Extraction

Extracting features from proteins is a challenge for identifying DNA-binding proteins. A suitable feature extraction algorithm can adequately represent the properties of the protein. We use four types of feature to describe a protein.

#### 4.1.1. MCD Feature

You et al. clustered the 20 amino acids into seven groups according to dipoles and volumes of side chains. These groups are {A, G, V}, {C}, {F, I, L, P}, {D, E}, {H, N, Q, W}, {K, R} and {M, S, T, Y}. A protein sequence “AVDCALSK” can be described as “11321476” via Multi-scale Continuous and Discontinuous descriptor (MCD) [52]. Then, above sequence was split into 10 local regions, which described multiple overlapping continuous and discontinuous interaction patterns. Composition (C), Transition (T) and Distribution (D) were calculated in each local region. The detailed descriptions of MCD algorithm can refer to You’s work [52]. The MCD feature was 882-dimentional vector.

#### 4.1.2. NMBAC Feature

Normalized Moreau–Broto Auto Correlation (NMBAC) [49,50] was proposed for extracting the sequence feature of membrane proteins. A protein sequence (string) can be represented as discrete numerical sequence via six physicochemical properties of Amino Acids (AA): including Hydrophobicity (H), Net Charge Index of Side Chains (NCISC), Solvent-Accessible Surface Area (SASA), Volumes of Side Chains of amino acids (VSC), Polarity (P1) and Polarizability (P2), respectively. The six physicochemical properties of amino acids are list in Table 5. To extract the feature of a protein X with *L*-length, the NMBAC feature is calculated by following equation:

(2) $$NMBAC\left(lag,j\right)=\frac{1}{\left(n-lag\right)}\sum_{i=1}^{n-lag}\left(X_{i,j}\times X_{i+lag,j}\right)$$

where *i* denote the position in the sequence, and i=1,2,…,n−lag. *j* is the type of physicochemical properties, j=1,2,…,6. lag∈[1,lg] is the gap between amino acids. lg is a parameter of maximum distance.

#### 4.1.3. PSSM-AB Feature

Position Specific Scoring Matrix (PSSM) contains evolutionary information of protein sequence. The PSSM of protein sequence is generated by PSI-BLAST [28]. PSSM is a L×20 matrix (*L* rows and 20 columns):

(3) $$PSSM={\left[\begin{array}{cccc} P_{1,1} & P_{1,2} & \cdots & P_{1,20}\\ P_{2,1} & P_{2,2} & \cdots & P_{2,20}\\ \vdots & \ddots & \vdots & \vdots \\ P_{L,1} & P_{L,2} & \cdots & P_{L,20} \end{array}\right]}_{L\times 20}$$

PSSM-AB extracts local average values of PSSM:

(4) $$PSSM-AB\left(k\right)=\frac{20}{L}\sum_{z=1}^{L/20}PSSM\left(z+\left(i-1\right)\times L/20,j\right)$$

where *k* is a linear index used to scan the cells of PSSM. i,j=1,2,…,20, k=j+20×(i−1). The PSSM-AB algorithm can extract the information of relationship between target residue and neighboring residues.

#### 4.1.4. PsePSSM Feature

PsePSSM [53] is an effective feature based on PSSM. PSSM ∈L×20 is standardized as following:

(5) $$\begin{array}{c} PSSM^{{}^{\prime }}\left(i,j\right)=\frac{PSSM(i,j)-mean(PSSM(i,*))}{STD(PSSM(i,*))}\\ i=1,2,\ldots ,L;j=1,2,\ldots ,20 \end{array}$$

where STD(PSSM(i,*)) denotes the standard deviation of the elements. mean(PSSM(i,*)) represents the mean of the elements that are located in the *i*-th row. * denotes the all elements of the *i*-th row. Then, we obtain the PsePSSM feature as the following:

(6) $$Pse\left(k\right)=\left\{\begin{array}{c} \frac{1}{L}\sum_{i=1}^{L}PSSM^{\prime }\left(i,j\right)\;k=1,\ldots ,20\\ \frac{1}{L-lag}\sum_{i=1}^{L-lag}{\left[PSSM^{\prime }\left(i,j\right)-PSSM^{\prime }\left(i+lag,j\right)\right]}^{2}\\ j=1,\ldots ,20;\;lag=1,\ldots ,15;\\ k=20+j+20\times (lag-1) \end{array}\right.$$

where *k* is index of feature vector and lag denotes the distance between one residue and its neighbors.

### 4.2. Multiple Kernel Learning

RBF is employed to construct 4 types of kernels via above features (including MCD, NMBAC, PSSM-AB and PsePSSM):

(7) $$K_{ij}=K\left(x_{i},x_{j}\right)=exp(-\gamma ∥x_{i}-x_{j}{∥}^{2}),\;i,j=1,2,\ldots ,N$$

where γ is the Gaussian kernel bandwidth. *N* is the number of samples. $x_{i}$ and $x_{j}$ are the feature vector of sample *i* and *j*. The 4 types of feature can be represented as a kernel set as: $\left\{K_{MCD},K_{NMBAC},K_{PSSM-AB},K_{PsePSSM}\right\}$.

The MKL algorithm combines multi-view features from different sources. Some kernels may have bias in the learning process. MKL can reduce bias of kernels by low weights. The optimal kernel $K_{train}^{*}$ is obtained as follows:

(8) $$K_{train}^{*}=\sum_{h=1}^{H}\omega_{h}K_{h},\;K^{*},K_{h}\in R^{N\times N}$$

where *H* denotes the number of basic kernels.

MKL algorithm [54] can estimate the optimal weights of kernels by minimize the distance between ideal kernel $K_{ideal}$ and optimal kernel $K_{train}^{*}$. The $K_{ideal}=y_{train}y_{train}^{T}\in R^{N\times N}$ denote the information of label space. $y_{train}\in R^{N\times 1}$ is the labels of training set. We hope that optimal kernel $K_{train}^{*}$ is close to the $K_{ideal}$ kernel:

(9a) $$\underset{\omega ,K^{*}}{min}\;∥K_{train}^{*}-K_{ideal}{∥}_{F}^{2}+\lambda {∥\omega ∥}_{F}^{2}$$

(9b) $$subject\;to\;K_{train}^{*}=\sum_{h=1}^{H}\omega_{h}K_{h},$$

(9c) $$\omega_{h}\geq 0,\;h=1,2,\ldots ,H,$$

(9d) $$\sum_{h=1}^{H}\omega_{h}=1$$

where ${∥X∥}_{F}^{2}=Trace\left(XX^{T}\right)$, λ is a regularization parameters, $\omega ={\left[\omega_{1},\omega_{2},\ldots ,\omega_{h}\right]}^{T}$ is the weights of kernels.

### 4.3. Fuzzy Kernel Ridge Regression

Kernel ridge regression is a method from statistics that implements a form of Regularized Least Squares (RLS). Given a training sample $x_{i},y_{i}$, i=1,2,…,N. *N*, $x_{i}$ and $y_{i}$ is the number of samples, feature vector and label. The RLS aims to find the minimum of the following function:

(10) $$J=\frac{C}{2}∥K_{train}\alpha -y_{train}{∥}^{2}+\frac{1}{2}{∥f∥}_{K}^{2}$$

where $K_{train}\in R^{N\times N}$ is the training kernel, *C* is the non-negative regular term. The solution of KRR is:

(11) $$\alpha ={\left(K_{train}+\frac{1}{C}I\right)}^{-1}y_{train}$$

In this paper, we present a Fuzzy Kernel Ridge Regression (FKRR) for classification. We need to minimize the sum of errors ($∥K_{train}\alpha -y_{train}{∥}^{2}$). The contribution of sample $x_{i}$ to the decision boundary should be proportional to its fuzzy membership value. The objective function is following function:

(12) $$J=\frac{C}{2}∥D\left(K_{train}\alpha -y_{train}\right){∥}^{2}+\frac{1}{2}{∥f∥}_{K}^{2}$$

where $D\in R^{N\times N}$ is a diagonal matrix whose element $D_{ii}$ ($0\leq D_{ii}\leq 1$) represents a fuzzy membership value for sample $x_{i}$.

We set ∂J/∂α=0 and the solution of α can be obtained as follows:

(13a) $$\begin{array}{c} \partial (\frac{C}{2}∥D\left(K_{train}\alpha -y_{train}\right){∥}^{2}+\frac{1}{2}∥f{∥}_{K}^{2})/\partial \alpha =0 \end{array}$$

(13b) $$\begin{array}{c} \partial (\frac{C}{2}∥D\left(K_{train}\alpha -y_{train}\right){∥}^{2}+\frac{1}{2}\alpha^{T}K_{train}\alpha )/\partial \alpha =0 \end{array}$$

(13c) $$\begin{array}{c} CK_{train}^{T}D^{T}\left(DK_{train}\alpha -Dy_{train}\right)+K_{train}\alpha =0 \end{array}$$

(13d) $$\begin{array}{c} CD^{2}\left(K_{train}\alpha -y_{train}\right)+\alpha =0 \end{array}$$

(13e) $$\begin{array}{c} \alpha ={\left(K_{train}+\frac{1}{C}D^{-2}I\right)}^{-1}y_{train} \end{array}$$

where $I\in R^{N\times N}$. So, the decision function is following:

(14a) $$\begin{array}{cc} y_{test} & =sign[K_{test}\alpha ] \end{array}$$

(14b) $$\begin{array}{c} =sign[K_{test}{\left(K_{train}+\frac{1}{C}D^{-2}I\right)}^{-1}y_{train}] \end{array}$$

where $y_{test}\in R^{M\times 1}$ is predictive labels. $K_{test}\in R^{M\times N}$ denotes the kernel of testing samples, *M* is the number of testing samples.

To compute fuzzy membership values of train samples, we employ the optimal kernels $K_{train}^{*}$ (training kernel) as following function:

(15) $$score_{t}=\frac{1}{N^{2}}\left(\sum_{y_{t}=y_{i}}K_{train}^{*}\left(x_{t},x_{i}\right)-\sum_{y_{t}\neq y_{i}}K_{train}^{*}\left(x_{t},x_{i}\right)\right)$$

where $score_{t}$ denotes the score of training point *t*. If a sample *t* has a larger score. This sample may has a greater contribution to model. We normalize scores into fuzzy membership values (0–1), as follows:

(16) $$D_{tt}=\frac{1}{1+exp(-score_{t})},\;t=1,2,\ldots ,N$$

## 5. Conclusions

FKRR-MVSF achieves better results on independent datasets (MCC: 0.676). Eliminating noise points can improve the predictive performance of the model. In the future, we aim to use other fuzzy membership functions to build fuzzy models for filtering the noise points. As pointed out in PseAAC-based methods [13,33,39,40,55,56,57,58,59,60], we will establish a web-server for our model. The related code and datasets can be download from: https://figshare.com/s/e80f1a96b7b7bbf8062b.

## Figures and Tables

**Table 1:** The detail information of two benchmark data sets.

Data Sets	PDB1075	PDB186
Positive	525	93
Negative	550	93
Total	1075	186

**Table 2:** The performance of different features on the PDB1075 dataset (Jackknife test).

Feature Type	Model	ACC	SN	Spec	MCC	AUC
MCD	KRR	0.7070	0.7086	0.7088	0.4139	0.7751
NMBAC	KRR	0.7284	0.7181	0.7382	0.4564	0.7857
PSSM-AB	KRR	0.7553	0.7695	0.7418	0.5113	0.8352
PsePSSM	KRR	0.7944	0.7905	0.7982	0.5886	0.8637
MW ^*a*^	KRR	0.8195	0.8362	0.8036	0.6398	0.8998
MKL	KRR	0.8214	0.8438	0.8000	0.6439	0.9032
MCD	SVM	0.7088	0.7345	0.6819	0.4171	0.7611
NMBAC	SVM	0.7116	0.6909	0.7333	0.4244	0.7706
PSSM-AB	SVM	0.7693	0.6981	0.8438	0.5467	0.8391
PsePSSM	SVM	0.7851	0.7472	0.8247	0.5731	0.8566
MW ^*a*^	SVM	0.8201	0.8232	0.8170	0.6421	0.9011
MKL	SVM	0.8299	0.8541	0.8057	0.6568	0.9101
MW ^*a*^	FKRR	0.8270	0.8533	0.8018	0.6554	0.9094
MKL	FKRR	0.8326	0.8571	0.8091	0.6664	0.9115

^*a*^ MW denotes combining kernels by the mean weights.

**Table 3:** Comparison between our method and other existing methods on the PDB1075 dataset (Jackknife test).

Methods	ACC (%)	MCC	SN (%)	Spec (%)
IDNA-Prot	75.40	0.50	83.81	64.73
DNAbinder	73.95	0.48	68.57	79.09
DNA-Prot	72.55	0.44	82.67	59.76
iDNAPro-PseAAC	76.56	0.53	75.62	77.45
IDNA-Prot|dis	77.30	0.54	79.40	75.27
Kmer1+ACC	75.23	0.50	76.76	73.76
Local-DPP	79.10	0.59	84.80	73.60
PseDNA-Pro	76.55	0.53	79.61	73.63
Adilina’s work	70.21	0.41	61.00	79.70
Our method (FKRR-MVSF)	83.26	0.67	85.71	80.91

**Table 4:** Compared with existing methods on the PDB186 dataset (Independent test).

Methods	ACC (%)	MCC	SN (%)	Spec (%)
IDNA-Prot	67.2	0.344	67.7	66.7
DNA-Prot	61.8	0.240	69.9	53.8
IDNA-Prot|dis	72.0	0.445	79.5	64.5
DNAbinder	60.8	0.216	57.0	64.5
DBPPred	76.9	0.538	79.6	74.2
Kmer1+ACC	71.0	0.431	82.8	59.1
iDNAPro-PseAAC	71.5	0.442	82.8	60.2
Local-DPP	79.0	0.625	92.5	65.6
Adilina’s work	82.3	0.670	95.0	69.9
MSFBinder (SVM)	81.7	0.640	89.3	74.2
Our method (FKRR-MVSF)	81.7	0.676	98.9	64.5

**Table 5:** The values of the 6 properties for twenty amino acids.

Amino Acid	H	VSC	P1	P2	SASA	NCISC
A	0.62	27.5	8.1	0.046	1.181	0.007187
C	0.29	44.6	5.5	0.128	1.461	−0.03661
D	−0.9	40	13	0.105	1.587	−0.02382
E	−0.74	62	12.3	0.151	1.862	0.006802
F	1.19	115.5	5.2	0.29	2.228	0.037552
G	0.48	0	9	0	0.881	0.179052
H	−0.4	79	10.4	0.23	2.025	−0.01069
I	1.38	93.5	5.2	0.186	1.81	0.021631
K	−1.5	100	11.3	0.219	2.258	0.017708
L	1.06	93.5	4.9	0.186	1.931	0.051672
M	0.64	94.1	5.7	0.221	2.034	0.002683
N	−0.78	58.7	11.6	0.134	1.655	0.005392
P	0.12	41.9	8	0.131	1.468	0.239531
Q	−0.85	80.7	10.5	0.18	1.932	0.049211
R	−2.53	105	10.5	0.291	2.56	0.043587
S	−0.18	29.3	9.2	0.062	1.298	0.004627
T	−0.05	51.3	8.6	0.108	1.525	0.003352
V	1.08	71.5	5.9	0.14	1.645	0.057004
W	0.81	145.5	5.4	0.409	2.663	0.037977
Y	0.26	117.3	6.2	0.298	2.368	0.023599
